# Phonological Awareness Mediates the Relationship between DCDC2 and Reading Performance with the Influence of Home Environment

**DOI:** 10.21203/rs.3.rs-2786924/v1

**Published:** 2023-05-10

**Authors:** Miao Li, Mellissa DeMille, Maureen Lovett, Joan Bosson-Heenan, Jan Frijters, Jeffrey Gruen

**Affiliations:** University of Houston/Harvard University; Yale University School of Medicine; The Hospital for Sick Children; Yale University School of Medicine; Brock University; Yale Medical School

**Keywords:** DCDC2-READ1, genetics, home environment, phonological skill, reading performance

## Abstract

Proficient reading requires critical phonological processing skill that interact with both genetic and environmental factors. However, the precise nature of the relationships between phonological processing and genetic and environmental factors are poorly understood. We analyzed data from the Genes, Reading and Dyslexia (GRaD) Study on 1,419 children ages 8 to 14 years from African-American and Hispanic-American family backgrounds living in North America. The analyses showed that phonological awareness mediated the relationship between *DCDC2*-READ1 and reading outcomes when parental education and socioeconomic status was low. The association between READ1 and reading performance is complex, whereby mediation by phonological awareness was significantly moderated by both parental education and socioeconomic status. These results show the importance of home environment and phonological skills when determining associations between READ1 and reading outcomes. This will be an important consideration in the development of genetic screening for risk of reading disability.

## 1. Introduction

Proficient reading is critical for success in school as well as lifetime earning potential. Children with low reading ability are more likely to live in poverty and have higher rates of unemployment as adults^1^. A great deal of research has been devoted to investigating the predictors of reading outcomes, including measures of individual word reading and reading comprehension, but the general consensus among reading researchers is that phonological processing skill, particularly phonological awareness, is a significant determinant of reading outcome^2,3^. Phonological processing refers to the use of phonological information in decoding written language^4^. Children with advanced phonological skills tend to have successful reading outcomes, whereas lower phonological skill is associated with reading difficultie^5,6^.

In addition to phonological skills, reading is also influenced by genetic factors. In studies of twins, the heritability of reading is high, ranging from .46 to .72^7,8^. However, heritability estimates are not uniformly high in molecular genetic studies of unrelated individuals assessed with single nucleotide polymorphisms (SNPs). While the differences in heritability estimates between family studies and SNP studies in psychiatric disorders is well known – frequently characterized as “missing heritability” – it is also likely that the relationship between genes and reading is indirect. Few studies rigorously address the connection between genes and reading outcomes. The prominent role of phonological skill in reading performance suggests that it may function as a mediator between specific genes and reading proficiency.

Approximately 18 genes have been associated with reading performance, but association with only 8 genes has been replicated three or more times: *CMIP*, *ATP2C2*, *FOXP2*, *ROBO1*, *DYXC1*, *KIAA0319*, *DCDC2*, and *CNTNAP2*^9^. Among them, only *KIAA0319* and *DCDC2* are located in the most replicated reading locus (DYX2; chromosome 6p22), and within both genes the peaks of association lie within regulatory features. We previously showed that we could identify children with a specific phonological deficit with variants of READ1, a regulatory element encoded within a known RD risk gene called *DCDC2*^10,11^.

Home environment is another factor related to reading and it can influence the relationship between genetics and reading. The variance due to genetic influence varies because home environment moderates genetic influences on reading outcomes^12^. Home environment may function as a condition on the mediating effect of phonological skill in the gene-reading linkage. In the present study, we aimed to examine the mediating role of phonological awareness between genes and reading. We were also interested in the moderating role of home environment to influence this mediation effect. By simultaneously considering the roles of phonological awareness and home environment, a moderated mediation model was tested to provide guidance in understanding how genetics affects reading performance.

### 1.1. The Connection between Phonological Processing and Reading Outcomes

It is well established that developmental and individual differences in phonological processing are causally related to reading ability in both longitudinal and experimental research^2,13^. Furthermore, deficit in phonological processing is a contributor to reading disability^14,15^.

Phonological awareness, a major component of phonological processing, refers to the sensitivity to and ability to manipulate sounds or sound structures of words. It is a powerful concurrent and longitudinal predictor of reading development^3,4,16^. According to the phonological deficit hypothesis, phonological awareness is a critical factor explaining difficulties in reading^17^. Interventions on phonological awareness training prove to be effective in improving reading performance of children with reading disability^5^. Thus, the connections between phonological awareness and rapid automatized naming and reading outcomes are well established and evidenced.

### 1.2. The Connection between Genetics and Reading Outcomes

The research showed significant and substantial genetic influences on reading performance^18^. For those who have difficulties in reading, research has shown that reading problems tend to run in families^19^. Twin studies research has shown large genetic influences on both word reading and reading comprehension from samples in Colorado, Ohio, Florida, England, Australia, and Scandinavia^7,20,21,22,23^. Although behavioral genetics studies can tell us whether reading is affected by genetic influences, they do not tell us which risk gene(s) can influence reading. This issue points to the high need of molecular genetics research.

Patterns of heritability toward genetic contribution and molecular genetics have identified risk genes that may cause reading difficulties^10, 24, 25, 26, 27^. Among the identified risk genes that are associated with reading diffi culties, *DCDC2*, located on chromosome 6p22, is the most replicated risk gene^10,11^. Research has shown that READ1 (regulatory element associated with dyslexia 1) is a regulatory element encoded in intron 2 of *DCDC2* and is a highly polymorphic complex tandem repeat with at least 40 alleles^10,11,28,29^. Among these alleles, RU2-Short that includes 6 or fewer copies of repeat unit 2 (alleles 4, 10, and 16 etc.) is considered a highly risk genetic variant of reading difficulties^30^. Clinical studies showed READ1 allele-specific association with severe reading and language impairment^29^. Nevertheless, the identified genes account for only a small portion of variance in reading difficulties^31^. The READ1 in the DYX2 locus should be further studied for its effects on reading performance.

### 1.3. The Connection between Genetics and Phonological Awareness

Previous studies have consistently found genetic influence on phonological awareness^8,32,33^. Furthermore, studies have shown that genes are responsible for the interrelations among phonological awareness and reading-related outcomes. For example, genetic influences were found to explain the comorbidity among phonological and orthographic skills^8^ or covariance between phonological awareness, rapid naming, and reading outcomes^33^.

### 1.4. The Genetic × Environment Influence on Reading

Home environment is crucial in literacy development. Parental education and socioeconomic status (SES) are important indicators of home environment^34,35,36,37,38^. Education is considered one of the most stable variables as it is usually established early in life and does not change over time. Parental education is highly correlated with children’s reading achievement^39,40^. SES is typically the most direct measure of family wealth and meta-analyses have demonstrated that SES is highly correlated with student achievement^40,41^.

Two models have been proposed to understand the relationship between genetic and environmental influences (G × E) on reading. One is the bioecological model which indicated that genetic influences should be greater in advantaged environment because genetic potential would be more fully realized in the supportive environments than in the poor environments^42^. The other is the diathesis-stress model which suggested that heritability should be greater in poor environments because deleterious genes may not be observed in more supportive environments. Both models are reasonable accounts of G × E interactions on reading. For example, individuals who carry the deleterious genes to put them as being reading disabled may experience the disadvantaged environment and such environmental triggers can activate deleterious genetic influences. Conversely, individuals who have the good genes may experience the supportive environments which may realize this genetic potential.

G × E interactions have been examined in reading abilities and disabilities^1,12,20, 43^ but the findings are mixed. For example, Kremen et al. (2005)^43^ found a shared environment × parental education interaction but not genetic × parental education interaction in a middle-aged men sample. This finding was confirmed by Taylor and Schatschneider’s (2010) study^20^. In Taylor and Schatschneider (2010), greater shared environmental influence than genetic influences were observed for first grade reading for the low-income group but not for the middle and high income groups. In contrast, Friend, DeFries, and Olson (2008)^1^ examined 545 identical and fraternal twins with at least one member of the pair who had the reading disability. They reported a G × E interaction and found that genetic influence was higher and environmental influence was lower among children whose parents had a high level of education. The heritability of low reading ability was significantly higher among children whose parents had higher levels of education, indicating that parental education moderated genetic influences on reading disability. Friend et al. (2009)^12^ further explored identical and fraternal twins with typically developing reading abilities from US and UK and reported that the heritability of high reading ability increased significantly with lower levels of parental education in both samples. Children whose parents had lower levels of education tend to have stronger genetic influence on their high reading ability. However, in a similarly aged sample, no moderating effects of parental education on genetic influences were found^44^. In addition, brain-behavior relationships critical for reading development are more pronounced in low SES environments^45^.

Overall, the findings from G × E interactions on reading ability are mixed. Much of the research on these topics has focused on behavioral genetics rather than molecular genetics and the G × E interaction research is mainly limited to twin studies. Further research is necessary to understand how genes interact with environment to affect reading ability. Ideally, a study testing this moderating effect should include molecular genetics with the identified genes that influence reading.

## 2. The Present Study

In the present study, we hypothesize that (1) phonological processing skill mediates the relationship between READ1 and reading outcomes including word reading and comprehension; and (2) that environmental factors moderate the mediation effect of phonological processing skill. To test these hypotheses, we analyzed data from the Genes, Reading and Dyslexia (GRaD) Study of 1,419 Hispanic-American and African-American participants, ages 8 years to 15 years. For phonological processing skills we used the Elision and Blending subtests of the Comprehensive Test of Phonological Processing (CTOPP)(Wagner *et al*., 1999). For reading outcomes we used the Woodcock-Johnson III - Letter-Word Identification and Word Attack (Woodcock *et al*., 2001) to assess word reading accuracy, Test of Word Reading Efficiency - Sight Word Efficiency and Phonetic Decoding Efficiency (TOWRE) (Torgesen *et al*., 1999) to assess word reading fluency, and the Standardized Reading Inventory - Passage Comprehension (SRI) (Newcomer, 1999) to assess reading comprehension. To assess the home environment, we used responses from the parental questionnaire. All subjects were genotyped for the READ1 allele, which were partitioned into three functional groups (see Methods). For the analysis, we tested a mediation model in which the relationship between READ1 genotype and reading outcomes was explained by phonological processing skills. Next, we investigated a moderation model in which the home environment factors moderated the relationship between READ1 and reading outcomes. Finally, we integrated the moderator into the mediation model and tested the moderated mediation model in which the strength of indirect (mediation) effect was conditional on the value of moderator (home environment factors).

## 3. Methods

### 3.1. Participants

There were 1,419 self-identified African-American and Hispanic-American children and adolescents who participated in this study. Their age range was from 8 to 15 years. This study was part of a larger, multi-site US and Canadian collaborative Genes, Reading, and Dyslexia (GRaD) project led by Yale University. The full set of sites included Albuquerque, NM; Baltimore, MD; Boston, MA; Boulder and Denver, CO; New Haven, CT; San Juan, PR; and Toronto, Canada. Participants with significant cognitive delays, behavioral problems, emotional/psychiatric disturbances, chronic neurologic conditions, and documented vision or hearing impairment were excluded.

### 3.2. Measures

#### 3.2.1. Woodcock-johnson Tests of Achievement, Third Edition (WJ-III)

Measures from the WJ-III included the Letter-Word Identification and Word Attack subtests (Woodcock *et al*., 2001). These measures were used to assess word reading accuracy. The WJ-III Letter Word Identification subtest asked the participant to read a list of increasingly complex single English words aloud. The Word Attack subtest required the participant to use knowledge of English phonology to decode a list of increasingly complex non-words or pseudowords in isolation. The total score for each subtest was the number of words read correctly. The standard score based upon age norms was then converted from the raw score. A composite score of both subtests was used to assess word reading accuracy in the study.

#### 3.2.2. Test of Word Reading Efficiency (TOWRE)

The TOWRE was a timed measure used to assess word reading fluency. It included subtests of single word reading (Sight Word Efficiency) and single pseudoword decoding (Phonemic Decoding Efficiency) (Torgesen *et al*., 1999). In the subtest of Sight Word Effiiency, the participant was required to read as many words as soon as possible within 45 seconds. In the subtest of Phonemic Decoding Efficiency, the participant was required to read as many pseudowords as soon as possible within 45 seconds. Standard scores for each subtest are the number of correctly read words or pseudowords within the time limit, relative to age norms. A composite score of both subtests was used to assess word reading fluency in the study.

#### 3.2.3. Standardized Reading Inventory, Second Edition (SRI).

The SRI (Newcomer, 1999) was used to acquire measures of Comprehension and Word Recognition Accuracy. This individually-administered contextual reading test consisted of 10 passages of increasing difficulty, ranging from pre-primer to an eighth-grade level. Accuracy is assessed during oral reading, followed by a series of questions to determine comprehension. Scores were obtained for word recognition accuracy and comprehension on each passage and then converted to standard scores based on age norms.

#### 3.2.4. Phonological Awareness

Phonological awareness was assessed using the Elision and Blending subtests of the Comprehensive Test of Phonological Processing (CTOPP) (Wagner *et al*., 1999). In the Blending subtest, phonological segments are synthesized to form a word. In the Elision task, a specified phonological segment is removed from a word, which forms a new word. The score for each subtest represents the number of correct items, converted to a standard score based on age norms. A composite score of both subtests was used to assess phonological awareness in the study.

#### 3.2.5. Home Environment Measures

Following consent and assent procedures, parents or guardians completed a questionnaire that covered family background, household resources, and the child’s education and health history. Parents or guardians reported years of formal education (ranging 6–18 years). Participation in a government assistance program was used to assess socioeconomic status (SES). For the families that received a government assistance program were coded as 1 and those without receiving such a program were coded as 0.

#### 3.2.6. Genotyping

Saliva was collected and DNA extracted using Oragene-DNA kits (DNA Genotek) following manufacturer protocols. SNP genotyping for rs2143340 was conducted as part of a larger Illumina HumanOmni2.5–8 bead chip, with genotyping calls screened for quality control measures. The call rate for rs2143340 in the GRaD sample was 0.983.

READ1 genotyping was conducted using PCR amplification and Sanger sequencing at the Yale W.M. Keck DNA Sequencing Facility using standard protocols as previously described (Li et al., 2018, 2020). Primer sequences and amplification protocol were as previously described (Powers et al., 2013). READ1 alleles were called from chromatograms using a custom program written in C++ (Dr. Yong Kong, available upon request). If the calling program identified errors, chromatograms were manually examined and deconvoluted for allele calling. The call rate for READ1 allele genotyping was 0.987.

The 2,445 bp microdeletion on 6p22, which encompasses the READ1 allele within breakpoints in intron 2 of *DCDC2*, was genotyped by allele specific PCR and agarose-gel electrophoresis. Primer sequences, amplification protocol, and gel electrophoresis for genotyping were as previously described^11^. The genotyping call rate for the microdeletion was 0.972.

#### 3.2.7. Functional Groups of READ1 Alleles

READ1 alleles were assigned to one of three groups: (1) RU1–1 alleles have only one copy of Repeat Unit 1 (RU1–1: alleles 2, 3, 9, 12, 25, 27); (2) RU2-Long alleles have two copies of RU1 and greater than seven copies of Repeat Unit 2 (RU2: alleles 5, 6, 13, 14, 19, 20, 22, 23); (3) RU2-Short is characterized by alleles that have fewer than six copies of Repeat Unit 2 (alleles 4, 10, 16, 21). Since we previously observed associations with RU2-Short in a group of African American and Hispanic American adolescents who had poor reading comprehension skills^30^, we primarily investigated the effect of RU2-Short in the current study.

### 3.3 Procedure

This study was approved by the Human Investigation Committee of Yale University and all of the institutional review boards of participating sites. Parental consent forms and child assent were collected before participation.

### 3.4. Data Analyses

We tested our hypotheses in three steps. First, we examined a mediation model in which the relationship between genetics and reading outcomes was explained by phonological processing skill. Second, we investigated a moderation model in which home environment factors moderated the relationship between genetics and reading outcomes. Finally, we integrated the moderator into the mediation model and tested the moderated mediation model in which the strength of the indirect (mediation) effect was conditional on the value of moderator (home environment) factors. Process software^46^ was used to investigate the moderation, mediation, and moderated mediation effects among reading, environmental, and genetic components.

## 4. Results

In the overall analyses (see Table 1), RU2-Short was negatively correlated with phonological awareness (*r* = − .09, *p* < .01) and reading comprehension (*r* = − .08, *p* < .01), and was positively correlated with SES (*r* = .08, *p* < .01; Table 1). Phonological awareness was significantly correlated with two home environment factors: parental education (*r* = .17, *p* < .01) and SES (*r* = − .13, *p* < .01) as well as three reading outcomes: word reading accuracy (*r* = .68, *p* < .01), word reading fluency (*r* = .59, *p* < .01), and reading comprehension (*r* = .56, *p* < .01). The three reading outcomes were also positively associated with each other: *r* = .84, *p* < .01 for the correlation between word reading accuracy and fluency, *r* = .68, *p* < .01 for the correlation between word reading accuracy and reading comprehension, and *r* = .62, *p* < .01 for the correlation between word reading fluency and reading comprehension.

### 4.1. Tests of Mediation

After showing correlation, we then tested for mediation with the three reading outcomes (see Table 2): (1) word reading accuracy assessed by a composite score of WJ-III Letter-Word Identification and Word Attack, (2) word reading fluency assessed by a composite score of TOWRE Sight Word Efficiency and Phonemic Decoding Efficiency, and (3) reading comprehension assessed by SRI Passage Comprehension. Phonological awareness and RU2-Short accounted for 69% of the variance when word reading accuracy was the dependent variable and RU2-Short was the independent variable. Although RU2-Short did not have a significant direct effect on word reading accuracy, it had a significant indirect effect (*b* = −1.74) through phonological awareness, with a bootstrapped 95% CI that did not cross zero around the indirect effect (−2.78, −0.68), indicating that phonological awareness had a significant mediation effect. When word reading fluency was the dependent variable, phonological awareness and RU2-Short accounted for 59% of the variance. Although RU2-Short did not have a significant direct effect on TOWRE, it had a significant indirect effect (*b* = −1.87) through phonological awareness with a bootstrapped 95% CI around the indirect effect (−2.94, −0.74) that did not cross zero, indicating that phonological awareness had a significant mediation effect. When reading comprehension was the dependent variable, phonological awareness and RU2-Short accounted for 56% of the variance. Although RU2-Short did not have a significant direct effect on reading comprehension, it had a significant indirect effect (*b* = − .40) with a bootstrapped 95% CI around the indirect effect (−.64, − .16) that did not cross zero, indicating that phonological awareness had a significant mediation effect. These analyses showed that phonological awareness mediated the effect of RU2-Short and all three of the reading outcomes.

### 4.2. Tests of Moderation

Next, we tested for moderation effects from parental education or SES (Table 3). Cross-product terms between parental education and word reading accuracy (*b* = .92, *p* < .01), parental education and word reading fluency (*b* = 1.00, *p* < .01), and between parental education and reading comprehension (*b* = .22, *p* = .01) were all significant. Cross-product terms between SES and word reading accuracy (*b* = −3.52, *p* = .02), and between SES and reading comprehension (*b* = −1.05, *p* = .01) were also significant.

### 4.3. Tests of Moderated Mediation

To examine whether the strength of the indirect mediation effect of RU2Short is conditional on the value of the either parental education or SES moderators, we then tested a moderated mediation model (Table 4). When word reading accuracy was the outcome, the interaction terms between RU2Short and moderators (parental education and SES) on PA were significant (*b* = 0.76, 95% CI [.18, 1.35]; *b* = −4.31, 95% CI [−7.42, −1.20]). Although RU2short did not have a direct effect on word reading accuracy, there were conditional indirect effects of three values of parental education (high, medium, low) and two values of SES (high and low) through PA. When parental education was low and medium, there was a significant indirect effect of RU2Short on word reading accuracy through PA (*b* = −3.39, 95% CI [−5.01, −1.78]; *b* = −1.93, 95% CI [−3.01, − .86]) but not when parent education was high (*b* = − .47, 95% CI [−2.01, 1.03]). When SES was low, there was a significant indirect effect of RU2short on word reading accuracy through PA (*b* = −2.91, 95% CI [−4.36, −1.54]), but not when SES was high (*b* = − .05, 95% CI [−1.53, 1.45]).

When word reading fluency was the outcome, the interaction terms between RU2Short and moderators on PA were significant (*b* = 0.77, 95% CI [.19, 1.36]; *b* = −4.41, 95% CI [−7.52, −1.30]). Although RU2short did not have a significant effect on word reading fluency, there were conditional indirect effects of RU2short on word reading fluency at three values of parental education and at two values of SES through PA. When parental education was low or medium, there was an indirect effect of RU2Short on word reading fluency through PA (*b* = −3.56, 95% CI [−5.25, −1.84]; *b* = −2.04, 95% CI [−3.07, − .91]), but not when parent education was high (*b* = − .51, 95% CI [−2.17, 1.07]). When SES was low, there was an indirect effect of RU2Short on word reading fluency through PA (*b* = − .67, 95% CI [−4.64, −1.61]), but not when SES was high (*b* = − .05, 95% CI [−1.65, 1.45]).

When reading comprehension was the outcome, the interaction terms between RU2Short and moderators on PA were significant (*b* = 0.77, 95% CI [.19, 1.36]; *b* = −4.34, 95% CI [−7.45, −1.23]). Although RU2Short did not have a direct and significant effect on reading comprehension, there were conditional indirect effects of RU2short on reading comprehension at three values of parental education and two values of SES through PA. When parental education was low or medium, there was an indirect effect of RU2Short on reading comprehension through PA (*b* = − .79, 95% CI [−1.18, − .42]; *b* = − .45, 95% CI [−.70, − .20]), but not when parent education was high (*b* = − .11, 95% CI [−.49, .26]). When SES was low, there was an indirect effect of RU2Short on reading comprehension through PA (*b* = − .67, 95% CI [−.99, − .35]) but not when SES was high (*b* = − .01, 95% CI [−.36, − .33]).

## 5. Discussion

In a study of mediation and moderation factors in 1,419 African-American and Hispanic-American children, we examined the influence of the genetic variant RU2-Short on word reading accuracy, word reading fluency, and reading comprehension. The results support a moderated mediation model, showing an indirect effect between RU2-Short and reading outcomes through phonological awareness, which was contingent on levels of parental education and SES.

### 5.1. Mediating Roles of Phonological Awareness

Although the heritability estimates for reading performance are not uniformly high, the variability in the results from previous studies may be partially explained by an indirect relationship between genetic factors and reading^47^. Longitudinal and intervention studies have shown that phonological awareness causally predicts reading outcomes^2,3,4,5^. Our results confirm the fully mediating role of phonological awareness in the connection between at least one risk gene (*DCDC2*) and the three reading outcomes that we tested. Other factors that likely contribute to the variability between studies include the generally small size of the cohorts, differences in assessments, and the study methods (for example, twins versus kinships)^1,7,12^.

### 5.2. Moderating Role of Home Environment

The nature of the interaction between genetic variants and environment on reading performance is also under-studied. Consistent with previous studies^20,43^, we show significant interactions between a well-known genetic risk variant (READ1) and home environment on reading outcomes, confirming that the relationship between genetics and phonological awareness can be adjusted by home environment factors. The strength of the indirect effect between risk genes and reading outcomes is conditional on the value of the home environment factors. When parental education and SES were low, there was a strong relationship between RU2-Short and reading performance. This supports the *diathesis-stress model*^48,49^, in which the heritability for reading is greater in a high-stress environment where stressors may lead to expression of risk genes. In contrast to the findings of Friend et al.^1,12^, we do not show that the genetic influence on RD is higher among children whose parents have a high level of education; this may be because Friend et al. did their studies in monozygotic and dizygotic twins, whereas we genotyped unrelated children.

### 5.3. The Moderated Mediation Model

A moderated mediation model could show that the effect of RU2-Short on reading outcomes is transmitted by phonological awareness, varying as a function of parental education and SES. In other words, phonological awareness mediates the relationship between genes and reading outcomes when parents have low education level but not when parents have medium and high education levels, and when SES is low, but less or perhaps not at all when SES is high. The connection between genes and reading performance is indirect through phonological awareness and is adjusted by different values of parental education and SES. While examining the influence of risk genes on reading, both cognitive and environmental factors need to be considered.

The present study broadens the scope of gene effects and presents a complex picture of how genes influence reading performance by considering the mediating role of phonological awareness, varying by parental education and SES. The finding is important because it suggests that in spite of a strong relationship between genes and phonological awareness, which in turn affects reading performance, the linkage between genes and phonological awareness is diminished when home environment is positive, and only becomes strong in more stressful environments.

## 6. Educational Implications

While reading ability continues to be a critical component of academic success, our results have several implications for education. The findings highlight the importance of phonological processing skill – particularly phonological awareness – as the main factor to explain the connection between genes and reading ability. In the classroom, teachers should still target the training of phonics to enhance students’ reading performance. Results from the present study of gene-by-environment interactions support the idea that risk genes tend to affect reading ability among children with parents having low education and in low SES families. Therefore, strategies to improve educational and home environments could be especially fruitful for children who carry risk genes for reading.

## 7. Limitations and Future Directions

Although our study is limited by its cross-sectional nature and a longitudinal design would have been more appropriate to test for mediation effects, it helps build the theoretical model of the moderated mediation. Furthermore, the mediator phonological awareness has been shown to be a causal factor of reading outcomes in both longitudinal and experimental designs^2,5,16^, making the mediation effect viable. Still, future research should examine and replicate our model with longitudinal and intervention data. In addition, we examined the contribution of only a single genetic risk variant, RU2-Short, to reading outcomes. Although RU2-Short is a functional genetic variant in the READ1 regulatory element for *DCDC2* and has been independently replicated, the correlations between it and all of the reading variables are small in magnitude though significant. Other genetic risk variants should also be investigated in future studies. Furthermore, our study focuses on two demographic groups (African-Americans and Hispanic-Americans) that have been under-represented in genetics research. To generalize our findings, future studies of more diverse populations, and larger cohorts, will need to be examined.

## Figures and Tables

**Figure 1:**
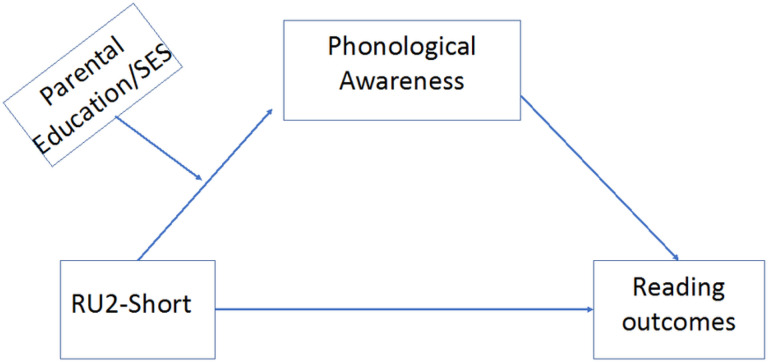
The proposed conceptual model

**Table 1:** Descriptive Statistics and Variable Intercorrelations

	Mean/SD	1	2	3	4	5	6	7
1. RU2-Short	.38/.49	--						
2. Phonological Awareness	92.85/14.35	− .09**	--					
3. Parental Education	13.60/2.92	.05	.17**	--				
4. Socioeconomic Status	.51/.50	.08**	− .13**	− .39**	--			
5. Word Reading Accuracy	94.50/13.75	− .04	.68**	.16**	− .14**	--		
6. Word Reading Fluency	92.65/16.75	− .05	.59**	.09**	− .08**	.84**	--	
7. Reading Comprehension	7.47/3.93	− .08**	.56**	.19**	− .18**	.68**	.62**	--

Note. Phonological Awareness = A composite score of CTOPP Elision and Blending; Word Reading Accuracy = A composite score of WJ-III Letter-Word Identification and Word Attack; Word Reading Fluency = A composite score of TOWRE Sight Word Efficiency and Phonemic Decoding Efficiency; Reading Comprehension = Standardized Reading Inventory

SD = Standard Deviation;

** *p* < .01.

**Table 2:** Regression Results for Mediation Effects

Mediator									
	Phonological Awareness			Phonological Awareness			Phonological Awareness		
	b	t	CI	b	t	CI	b	t	CI
RU2-Short	−2.64**	−3.30	[−4.20, −1.07]	−2.69**	−3.38	[−4.26, −1.13]	−2.65**	−3.32	[−4.21, −1.08]
Dependent Variables									
	Word Reading Accuracy			Word Reading Fluency			Reading Comprehension		
RU2-Short	.48	.86	[−.62, 1.59]	.12	.16	[−1.37, 1.62]	− .26	− 1.40	[−.61, .10]
Phonological Awareness	.66**	34.58	[.63, .70]	.69**	26.76	[.64, .74]	.15**	24.32	[.14, .16]
Indirect Effect									
Phonological Awareness	−1.74		[−2.78, − .68]	−1.87		[−2.94, − .74]	− .40		[−.64, − .16]

Note. Phonological Awareness = A composite score of CTOPP Elision and Blending; Word Reading Accuracy = A composite score of WJ-III Letter-Word Identification and Word Attack; Word Reading Fluency = A composite score of TOWRE Sight Word Efficiency and Phonemic Decoding Efficiency; Reading Comprehension = Standardized Reading Inventory

** p < .01

**Table 3:** Regression Results for Moderation Effects

Predictor	*b*	*SE*	*t*	*p*
	Word Reading Accuracy			
RU2Short	−14.19	3.97	−3.58	.00
Parental Education	.50	.16	3.14	.00
RU2Short × Parental Education	.92	.28	3.24	.00
	Word Reading Fluency			
RU2Short	−15.68	4.89	−3.21	.00
Parental Education	.27	.19	1.37	.17
RU2Short × Parental Education	1.00	.35	2.85	.00
	Reading Comprehension			
RU2Short	−3.67	1.13	−3.25	.00
Parental Education	.21	.05	4.77	.00
RU2Short × Parental Education	.22	.08	2.67	.01
	Word Reading Accuracy			
RU2Short	.91	1.11	.82	.41
SES	−2.43	.94	−2.59	.01
RU2Short × SES	−3.52	1.53	−2.30	.02
	Word Reading Fluency			
RU2Short	.32	1.36	.23	.82
SES	−1.66	1.15	−1.44	.15
RU2Short × SES	− .3.52	1.87	−1.88	.06
	Reading Comprehension			
RU2Short	.02	.31	.07	.95
SES	− .99	.26	−3.76	.00
RU2Short × SES	−1.05	.43	−2.45	.01

Note. Word Reading Accuracy = A composite score of WJ-III Letter-Word Identification and Word Attack; Word Reading Fluency = A composite score of TOWRE Sight Word Efficiency and Phonemic Decoding Efficiency; Reading Comprehension = Standardized Reading Inventory; SES = Socioeconomic Status

**Table 4:** Regression Results for Moderated Mediation Effects

	Mediator								
	Phonological Awareness			Phonological Awareness			Phonological Awareness		
	b	t	CI	b	t	CI	b	t	CI
RU2-Short	−13.33**	−3.20	[−21.51, −5.15]	−13.52**	4.17	[−21.70, −5.34]	−13.49**	−3.24	[−21. −5.31
PE	.67**	4.04	[.34, .99]	.67**	.17	[.35, 1.00]	.67**	4.05	[.35, 1.00
RU2-Short × PE	.76**	2.56	[.18, 1.35]	.77**	.30	[.19, 1.36]	.77**	2.60	[.19, 1.36
	Dependent Variables								
	Word Reading Accuracy			Word Reading Fluency			Reading Comprehension		
RU2-Short	.36	.62	[−.78, 1.50]	.04	.05	[−1.52, 1.59]	− .25	−1.31	[−.63 .13]
PA	.66**	33.42	[.62, .69]	.68**	25.35	[.63, .73]	.15**	23.35	[.14, .16]
Conditional Indirect Effect									
Low PE	−3.39		[−5.01, −1.78]	−3.56		[−5.25, −1.84]	− .79		[−1.1 − .42
Medium PE	−1.93		[−3.01, − .86]	−2.04		[−3.07, − .91]	− .45		[−.70 − .20
High PE	− .47		[−2.01, 1.03]	− .51		[−2.17, 1.07]	− .11		[−.49 .26]
	Mediator								
	Phonological Awareness			Phonological Awareness			Phonological Awareness		
	b	t	CI	b	t	CI	b	t	CI
RU2-Short	− .08	− .07	[−2.33, −2.18]	− .08	− .07	[−2.33, −2.18]	− .08	− .07	[−2.33, 2.18]
SES	−2.12*	−2.18	[−4.04, − .21]	−2.08*	−2.14	[−3.99, − .17]	−2.09*	−2.14	[−3.99, − .18]
RU2-Short × SES	−4.31**	−2.72	[−7.42, −1.20]	−4.41**	−2.78	[−7.52, −1.30]	−4.34**	−2.74	[−7.45, −1.23]
	Dependent Variables								
	Word Reading Accuracy			Word Reading Fluency			Reading Comprehension		
RU2-Short	.48	.86	[−.62, 1.59]	.12	.16	[−1.37, 1.62]	− .26	−1.40	[−.61, .10]
PA	.66**	34.58	[.63, .70]	.69**	26.76	[.64, .74]	.15**	24.32	[.14, .16]
Conditional Indirect Effect									
High SES	− .05		[−1.53, 1.45]	− .05		[−1.65, 1.45]	− .01		[−.36, .33]
Low SES	−2.91		[−4.36, −1.54]	−3.11		[−4.64, −1.61]	− .67		[−.99, − .35]

Note. Phonological Awareness = A composite score of CTOPP Elision and Blending; Word Reading Accuracy = A composite score of WJ-III Letter-Word Identification and Word Attack; Word Reading Fluency = A composite score of TOWRE Sight Word Efficiency and Phonemic Decoding Efficiency; Reading Comprehension = Standardized Reading Inventory; PE = Parental Education; SES = Socioeconomic Status;

** *p* < .01

## Data Availability

The data that support the findings of this study are available from the corresponding author upon reasonable request.

## References

[1] Friend A, DeFries JC, Olson RK (2008) Parental education moderates genetic influences on reading disability. Psychological Science 19(11): 1124–1130. 19076484 10.1111/j.1467-9280.2008.02213.x PMC2605635

[2] Wagner RK, Torgesen JK (1987) The nature of phonological processing and its causal role in the acquisition of reading skills. Psychological bulletin 101(2):192–212.

[3] Torgesen JK, Wagner RK, Rashotte CA (1994) Longitudinal studies of phonological processing and reading. Journal of learning disabilities 27(5):276–86. 8006506 10.1177/002221949402700503

[4] Adams MJ (1990) Beginning to read: Thinking and learning about print. Cambridge, MA: MIT Press.

[5] Lovett MW, Lacerenza L, Borden SL, Frijters JC, Steinbach KA, De Palma M (2000) Components of effective remediation for developmental reading disabilities: Combining phonological and strategy-based instruction to improve outcomes. Journal of educational psychology 92(2):263–283.

[6] Snowling MJ, Hulme CE (2005) The science of reading: A handbook. Blackwell Publishing.

[7] Byrne B, Coventry WL, Olson RK, Samuelsson S, Corley R, Willcutt EG, (2009) Genetic and environmental influences on aspects of literacy and language in early childhood: Continuity and change from preschool to Grade 2. Journal of Neurolinguistics 22(3): 219–236. 20161176 10.1016/j.jneuroling.2008.09.003 PMC2724015

[8] Gayán J, Olson RK (2001) Genetic and environmental influences on orthographic and phonological skills in children with reading disabilities. Developmental neuropsychology 20(2):483–507. 11892949 10.1207/S15326942DN2002_3

[9] Landi N, Perdue MV (2019) Neuroimaging genetics studies of specific reading disability and developmental language disorder: A review. Lang Linguist Compass 13(9):e12349. 31844423 10.1111/lnc3.12349 PMC6913889

[10] Meng H, Smith SD, Hager K, Held M, Liu J, Olson RK, Pennington BF, DeFries JC, Gelernter J, O’Reilly-Pol T, Somlo S (2005) DCDC2 is associated with reading disability and modulates neuronal development in the brain. Proceedings of the National Academy of Sciences 102(47):17053–17058. 10.1073/pnas.0508591102 PMC1278934 16278297

[11] Powers NR, Eicher JD, Butter F, Kong Y, Miller LL, Ring SM, (2013) Alleles of a polymorphic ETV6 binding site in DCDC2 confer risk of reading and language impairment. The American Journal of Human Genetics 93(1):19–28. 23746548 10.1016/j.ajhg.2013.05.008 PMC3710765

[12] Friend A, DeFries JC, Olson RK, Pennington B, Harlaar N, Byrne B, Samuelsson S, Willcutt EG, Wadsworth SJ, Corley R, Keenan JM. (2009) Heritability of high reading ability and its interaction with parental education. Behavior genetics 39(4):427–36. 19296213 10.1007/s10519-009-9263-2 PMC3387983

[13] Wagner RK, Torgesen JK, Rashotte CA (1999) CTOPP: Comprehensive test of phonological processing. Austin, TX: Pro-ed.

[14] Scarborough H. S. (1998). Predicting the future achievement of second graders with reading disabilities: Contributions of phonemic awareness, verbal memory, rapid naming, and IQ. Annals of Dyslexia, 48, 115–136.

[15] Vellutino F. R., Fletcher J. M., Snowling M. J., & Scanlon D. M. (2004). Specific reading disability (dyslexia): What have we learned in the past four decades? Journal of child psychology and psychiatry, 45(1), 2–40. 14959801 10.1046/j.0021-9630.2003.00305.x

[16] Torgesen JK, Wagner RK, Rashotte CA, Burgess S, Hecht S (1997) Contributions of phonological awareness and rapid automatic naming ability to the growth of word-reading skills in second-to fifth-grade children. Scientific studies of reading 1(2):161–185.

[17] Ramus F. (2003). Developmental dyslexia: specific phonological deficit or general sensorimotor dysfunction? Current opinion in neurobiology, 13(2), 212–218. 12744976 10.1016/s0959-4388(03)00035-7

[18] Keenan J. M., Betjemann R. S., Wadsworth S. J., DeFries J. C., & Olson R. K. (2006). Genetic and environmental influences on reading and listening comprehension. Journal of research in reading, 29(1), 75–91.

[19] Pennington B. F., & Olson R. K. (2005). Genetics of Dyslexia. In Snowling M. J. & Hulme C. (Eds.), The science of reading: A handbook (pp. 453–472). Blackwell Publishing.

[20] Taylor J, Schatschneider C (2010) Genetic influence on literacy constructs in kindergarten and first grade: Evidence from a diverse twin sample. Behavior genetics 40(5):591–602. 20563747 10.1007/s10519-010-9368-7 PMC3529359

[21] Christopher M. E., Hulslander J., Byrne B., Samuelsson S., Keenan J. M., Pennington B., … & Olson R. K. (2013). The genetic and environmental etiologies of individual differences in early reading growth in Australia, the United States, and Scandinavia. Journal of Experimental Child Psychology, 115(3), 453–467. 23665180 10.1016/j.jecp.2013.03.008 PMC3661747

[22] Olson R. K., Hulslander J., Christopher M., Keenan J. M., Wadsworth S. J., Willcutt E. G., … & DeFries J. C. (2013). Genetic and environmental influences on writing and their relations to language and reading. Annals of dyslexia, 63, 25–43. 21842316 10.1007/s11881-011-0055-z PMC3218215

[23] Logan J. A., Hart S. A., Cutting L., Deater-Deckard K., Schatschneider C., & Petrill S. (2013). Reading development in young children: Genetic and environmental influences. Child development, 84(6), 2131–2144. 23574275 10.1111/cdev.12104 PMC3773299

[24] Cope N., Harold D., Hill G., Moskvina V., Stevenson J., Holmans P., … & Williams J. (2005). Strong evidence that KIAA0319 on chromosome 6p is a susceptibility gene for developmental dyslexia. The American Journal of Human Genetics, 76(4), 581–591. 15717286 10.1086/429131 PMC1199296

[25] Eicher J. D., Powers N. R., Miller L. L., Mueller K. L., Mascheretti S., Marino C., … & Gruen J. R. (2014). Characterization of the DYX2 locus on chromosome 6p22 with reading disability, language impairment, and IQ. Human Genetics, 133, 869–881. 24509779 10.1007/s00439-014-1427-3 PMC4053598

[26] Pinel P., Fauchereau F., Moreno A., Barbot A., Lathrop M., Zelenika D., … & Dehaene S. (2012). Genetic variants of FOXP2 and KIAA0319/TTRAP/THEM2 locus are associated with altered brain activation in distinct language-related regions. Journal of Neuroscience, 32(3), 817–825. 22262880 10.1523/JNEUROSCI.5996-10.2012 PMC6621137

[27] Skeide M. A., Kraft I., Müller B., Schaadt G., Neef N. E., Brauer J., … & Friederici A. D. (2016). NRSN1 associated grey matter volume of the visual word form area reveals dyslexia before school. Brain, 139(10), 2792–2803. 27343255 10.1093/brain/aww153

[28] Meng H., Powers N. R., Tang L., Cope N. A., Zhang P. X., Fuleihan R., … & Gruen J. R. (2011). A dyslexia-associated variant in DCDC2 changes gene expression. Behavior genetics, 41, 58–66. 21042874 10.1007/s10519-010-9408-3 PMC3053575

[29] Powers N. R., Eicher J. D., Miller L. L., Kong Y., Smith S. D., Pennington B. F., … & Gruen J. R. (2016). The regulatory element READ1 epistatically influences reading and language, with both deleterious and protective alleles. Journal of Medical Genetics, 53(3), 163–171. 26660103 10.1136/jmedgenet-2015-103418 PMC4789805

[30] Li M, Malins JG, DeMille MM, Lovett MW, Truong DT, Epstein K, Lacadie C, Mehta C, Bosson-Heenan J, Gruen JR, Frijters JC (2018) A molecular-genetic and imaging-genetic approach to specific comprehension diffi culties in children. npj Science of Learning 21(3). 10.1038/s41539-018-0034-9 PMC6249284 30631481

[31] Newbury D. F., Monaco A. P., & Paracchini S. (2014). Reading and language disorders: the importance of both quantity and quality. Genes, 5(2), 285–309. 24705331 10.3390/genes5020285 PMC4094934

[32] Hohnen B., & Stevenson J. (1999). The structure of genetic influences on general cognitive, language, phonological, and reading abilities. Developmental Psychology, 35(2), 590. 10082029 10.1037//0012-1649.35.2.590

[33] Petrill S. A., Deater-Deckard K., Thompson L. A., DeThorne L. S., & Schatschneider C. (2006). Genetic and environmental effects of serial naming and phonological awareness on early reading outcomes. Journal of educational psychology, 98(1), 112. 19444324 10.1037/0022-0663.98.1.112 PMC2681098

[34] Burgess SR, Hecht SA, Lonigan CJ (2002) Relations of the home literacy environment (HLE) to the development of reading-related abilities: A one-year longitudinal study. Reading Research Quarterly 37(4): 408–426.

[35] Frijters JC, Barron RW, Brunello M (2000) Direct and mediated influences of home literacy and literacy interest on prereaders’ oral vocabulary and early written language skill. Journal of Educational psychology 92(3):466–477.

[36] Sénéchal M, LeFevre JA (2002) Parental involvement in the development of children’s reading skill: A five-year longitudinal study. Child Development 73(2):445–460. 11949902 10.1111/1467-8624.00417

[37] Sénéchal M, LeFevre JA (2014) Continuity and change in the home literacy environment as predictors of growth in vocabulary and reading. Child Development. 85(4):1552–1568. 24467656 10.1111/cdev.12222

[38] Smith TE, Graham PB (1995) Socioeconomic stratification in family research. Journal of Marriage and the Family 1:930–940.

[39] Sirin S. R. (2005). Socioeconomic status and academic achievement: A meta-analytic review of research. Review of educational research, 75(3), 417–453.

[40] White K. R. (1982). The relation between socioeconomic status and academic achievement. Psychological bulletin, 91(3), 461.

[41] Hauser R. M. (1994). Measuring socioeconomic status in studies of child development. Child development, 65(6), 1541–1545. 7859541 10.1111/j.1467-8624.1994.tb00834.x

[42] Bronfenbrenner U., & Ceci S. J. (1994). Nature-nuture reconceptualized in developmental perspective: A bioecological model. Psychological review, 101(4), 568. 7984707 10.1037/0033-295x.101.4.568

[43] Kremen WS, Jacobson KC, Xian H, Eisen SA, Waterman B, Toomey R, Neale MC, Tsuang MT, Lyons MJ (2005) Heritability of word recognition in middle-aged men varies as a function of parental education. Behavior Genetics 35(4):417–433. 15971023 10.1007/s10519-004-3876-2

[44] Kirkpatrick R. M., Legrand L. N., Iacono W. G., & McGue M. (2011). A twin and adoption study of reading achievement: Exploration of shared-environmental and gene–environment-interaction effects. Learning and individual differences, 21(4), 368–375. 21743785 10.1016/j.lindif.2011.04.008 PMC3130536

[45] Noble K. G., Wolmetz M. E., Ochs L. G., Farah M. J., & McCandliss B. D. (2006). Brain–behavior relationships in reading acquisition are modulated by socioeconomic factors. Developmental science, 9(6), 642–654. 17059461 10.1111/j.1467-7687.2006.00542.x

[46] Hayes A. F. (2017). Introduction to mediation, moderation, and conditional process analysis: A regression-based approach. Guilford publications.

[47] Nation K (2006) Reading and genetics: An introduction. Journal of Research in Reading 29: 1–10.

[48] Zubin J., & Spring B. (1977). Vulnerability: A new view of schizophrenia. Journal of Abnormal Psychology, 86, 103–126. 858828 10.1037//0021-843x.86.2.103

[49] Scarr S. (1992). Developmental theories for the 1990s: Development and individual differences. Child Development, 63, 1–19. 1343618

